# Predictive capacity of immune‐related adverse events and cytokine profiling in neoadjuvant immune checkpoint inhibitor trials for head and neck squamous cell carcinoma

**DOI:** 10.1002/cam4.7370

**Published:** 2024-06-07

**Authors:** Angela E. Alnemri, Sruti Tekumalla, Annie E. Moroco, Ioannis Vathiotis, Madalina Tuluc, Stacey Gargano, Tingting Zhan, David M. Cognetti, Joseph M. Curry, Athanassios Argiris, Alban Linnenbach, Andrew P. South, Larry A. Harshyne, Jennifer M. Johnson, Adam J. Luginbuhl

**Affiliations:** ^1^ Department of Otolaryngology – Head & Neck Surgery Thomas Jefferson University Philadelphia Pennsylvania USA; ^2^ Department of Medical Oncology Thomas Jefferson University Philadelphia Pennsylvania USA; ^3^ Department of Pathology Thomas Jefferson University Philadelphia Pennsylvania USA; ^4^ Department of Biostatistics Thomas Jefferson University Philadelphia Pennsylvania USA; ^5^ Department of Dermatology and Cutaneous Biology Thomas Jefferson University Philadelphia Pennsylvania USA; ^6^ Department of Pharmacology, Physiology and Cancer Biology Thomas Jefferson University Philadelphia Pennsylvania USA; ^7^ Department of Microbiology & Immunology Thomas Jefferson University Philadelphia Pennsylvania USA

**Keywords:** cytokines, head and neck neoplasms, immunotherapy, tumor biomarkers

## Abstract

**Objectives:**

Certain low‐level immune‐related adverse events (irAEs) have been associated with survival benefits in patients with various solid tumors on immune checkpoint inhibitors (ICIs). We aimed to investigate the association between irAEs and response to neoadjuvant ICIs in patients with head and neck squamous cell carcinoma (HNSCC) and to identify differences in circulating cytokine levels based on irAE status.

**Methods:**

This was a retrospective cohort study including three neoadjuvant clinical trials from July 2017 to January 2022: NCT03238365 (nivolumab ± tadalafil), NCT03854032 (nivolumab ± BMS986205), NCT03618654 (durvalumab ± metformin). The presence and type of irAEs, pathologic treatment response, and survival were compared. Canonical linear discriminant analysis (LDA) was performed to identify combinations of circulating cytokines predictive of irAEs using plasma sample multiplex assay.

**Results:**

Of 113 participants meeting inclusion criteria, 32 (28.3%) developed irAEs during treatment or follow‐up. Positive p16 status was associated with irAEs (odds ratio [OR] 2.489; 95% CI 1.069–6.119; *p* = 0.043). irAEs were associated with pathologic treatment response (OR 3.73; 95% CI 1.34–10.35; *p* = 0.011) and with higher OS in the combined cohort (HR 0.319; 95% CI 0.113–0.906; *p* = 0.032). Patients with irAEs within the nivolumab cohort had significant elevations of select cytokines pre‐treatment. Canonical LDA identified key drivers of irAEs among all trials, which were highly predictive of future irAE status.

**Conclusions:**

irAEs are associated with response to neoadjuvant ICI therapy in HNSCC and can serve as clinical indicators for improved clinical outcomes. irAEs can be predicted by concentrations of several circulating cytokines prior to treatment.

## 1 BACKGROUND

Head and neck squamous cell carcinoma (HNSCC) is the most common malignancy in the head and neck. While the mainstay of treatment for HNSCC involves chemotherapy, radiation, and/or surgery, its immunological features suggest that it has a high potential for responding to immunotherapeutic agents such as immune checkpoint inhibitors (ICIs). Tumor cells use various mechanisms to evade immunosurveillance, including activation of immune checkpoint pathways to suppress antitumor responses. ICIs combat this by interrupting co‐inhibitory signaling pathways and promoting the elimination of tumor cells through immune‐mediated pathways.^1^

The use of ICIs is associated with a class of side effects known as immune‐related adverse events (irAEs) across all tumor types. The frequency of these reactions varies based on the ICI used, use of monotherapy or combination therapy, patient factors (i.e., presence of autoimmune disorders, certain medications such as steroids), and to a lesser extent, the tumor type being treated. These reactions can range from mild, transient reactions to severe reactions leading to acute and chronic morbidity or death.^2^, ^3^ irAEs can occur in almost any organ but are most commonly present as dermatitis, endocrinopathies, or colitis.^4^ Current literature has linked irAEs to improved oncologic outcomes in various malignancies treated with ICIs, most notably melanoma and non‐small cell lung cancer (NSCLC), with few studies supporting a unified predictive role for irAEs in HSNCC.^5^, ^6^, ^7^, ^8^, ^9^, ^10^, ^11^, ^12^, ^13^, ^14^, ^15^, ^16^, ^17^, ^18^

irAEs are thought to represent bystander effects from activated T‐cells and/or disinhibition of immune checkpoints that protect against autoimmunity.^1^, ^5^ This suggests that variations in T‐cell function among patients could contribute to the development of irAEs. Research into predictive biomarkers for irAEs is still in its infancy. Recent studies have begun to investigate the association between circulating cytokines and irAEs. Cytokines are soluble mediators that regulate host immune activity. In the context of cancer, one of their many roles is the promotion of immune cell infiltration into the tumor microenvironment (TME).^19^, ^20^ Consequently, circulating cytokine levels could affect how a patient reacts to immunotherapy and act as predictive biomarkers for the development of irAEs.

In this study, we explore the relationship between irAEs and response to immunotherapy in patients with HNSCC who underwent treatment in one of three neoadjuvant ICI‐based clinical trials. We also aim to investigate the association between baseline circulating cytokines and the development of irAEs. We hypothesize that the development of irAEs will correlate with improved oncologic outcomes in patients with HNSCC treated with neoadjuvant ICIs. Further, we hypothesize that there is differential baseline expression of cytokines in patients with irAEs that may be helpful in predicting irAE risk.

## 2 METHODS

### 2.1 Study population and design

Patients with HNSCC enrolled in one of three neoadjuvant ICI‐based clinical trials conducted at Thomas Jefferson University (with one trial also conducted Vanderbilt University) between May 2017 and January 2022 were included. Patients were excluded on the grounds of trial withdrawal before first treatment or incomplete data. All clinical trials had unique full approval by the Thomas Jefferson University Institutional Review Board. This retrospective study was determined to be exempt from review (IRB #21E.431).

#### 2.1.1 Window of opportunity trial of nivolumab and tadalafil in patients with squamous cell carcinoma of the head and neck (NCT03238365) (Nivo±Tad)

This was an investigator‐initiated, two‐arm multi‐institutional (Thomas Jefferson University and Vanderbilt University) randomized trial involving patients with newly diagnosed and resectable HNSCC of any stage by the American Joint Committee on Cancer Criteria (AJCC) 8th edition. Subjects were randomized 1:1 to receive the programmed death‐1 (PD‐1) inhibitor nivolumab (Bristol‐Myers Squibb, New York, NY) alone or nivolumab plus tadalafil (Eli Lilly, Indianapolis, IN) with stratification for human papillomavirus (HPV) status. On days 3 (±2) and 17 (±2), subjects in both cohorts received nivolumab 240 mg intravenously, with those in the combination cohort also receiving tadalafil 10 mg orally daily for 4 weeks beginning on day 3 (±2). All patients underwent definitive surgical resection at approximately 4 weeks.^21^

#### 2.1.2 Window of opportunity trial of nivolumab and BMS986205 in patients with squamous cell carcinoma of the head and neck (NCT03854032) (Nivo±IDO)

This was a phase I investigator‐initiated, two‐arm randomized trial. Patients with any‐stage (AJCC 8th edition) resectable HNSCC were included. Subjects were randomized 3:1 to receive nivolumab + BMS986205 (Indoleamine 2,3‐dioxygenase [IDO] inhibitor) or nivolumab alone. Patients in the nivolumab + BMS986205 arm received BMS986205 100 mg oral daily for 28 days beginning on day 3 (±3). Patients in both arms received 1 dose of nivolumab 480 mg IV at day 10 (±3). Tumor radiographic response at the primary tumor site and regional lymph nodes were assessed at approximately 5 weeks. Non‐responders underwent definitive surgical resection at this time. Responders repeated their treatment for an additional 4 weeks based on initial randomization arm followed by definitive surgical resection.

#### 2.1.3 Window of opportunity for durvalumab (MEDI4736) plus metformin trial in squamous cell carcinoma of the head and neck (NCT03618654) (Durva±Met)

This was a phase I investigator‐initiated, two‐arm randomized trial. Patients with any‐stage (AJCC 8th edition) resectable HNSCC were included. Subjects were randomized 3:1 to the programmed death‐ligand 1 (PD‐L1) inhibitor durvalumab (Medimmune/AstraZeneca) + metformin or durvalumab alone. Patients in the durvalumab + metformin arm received a four‐week supply of metformin to begin on day 1 (±2). Subjects in this arm began with metformin 500 mg oral daily for 3 days, titrated to 500 mg twice daily for an additional 3 days. If tolerated, the dose was again increased to 1000 mg twice daily after day 6. Patients maintained the maximum tolerated dose until the day prior to surgery. All subjects received 1500 mg durvalumab (MEDI4736) via IV infusion on day 3 (±2). Definitive surgical resection was performed approximately 4 weeks.

### 2.2 Outcome measures

Each trial prospectively collected adverse events classified as immune‐related, treatment‐related, or non‐related to the intervention by the study principal investigator. For this study, irAEs of any grade were included. Additional data retrospectively collected included age, sex, race, smoking status, disease site, tumor staging, p16 status, and follow‐up data, including timing of irAE onset, survival data, and adjuvant treatment, which was determined by current standard of care guidelines and discussion at our Multidisciplinary Tumor Board. p16 status was used as a surrogate for HPV status and was determined by immunohistochemistry staining of tumor samples for p16. For all trials, clinical staging was determined by clinical exam, computed tomography, and 18F‐fluorodeoxyglucose‐positron emission tomography/computed tomography scans at enrollment utilizing the AJCC 8th edition.

### 2.3 Assessment of pathologic treatment response

All trials involved definitive cancer resection. Pathologic specimens from the day of surgical resection were independently graded by two experienced head and neck pathologists (MT, SG) utilizing scanned digital slides. All slides with tumor (primary and all lymph nodes) included in the analysis provided a pathologic treatment effect (pTE). Change in pTE (%) was equal to Areas of Treatment Effect divided by Total Tumor Surface Area. Histologic criteria constituting pTE included areas of macrophage reaction, multinucleated giant cells and granulomas, fibrosis, and chronic inflammation adjacent to residual tumor nests. Based on pTE of the post‐treatment specimen, patients were classified as responders and non‐responders.^22^ In Nivo±Tad and Nivo±IDO, responders were defined as having a pTE% ≥ 20% and non‐responders a pTE% of < 20% based on the average pTE at the primary site and lymph nodes. In Durva±Met, responders were defined as having a pTE% > 10% and non‐responders a pTE% ≤ 10% at either the primary site or lymph nodes. Within Durva±Met, pTE was not quantified and was instead provided as categorical datapoints based on pTE% parameters. These criteria were endpoints set by independent parameters of each trial design.

### 2.4 Cytokine analysis

Peripheral whole blood samples were taken at the time of study recruitment (pre‐treatment) and following completion of the neoadjuvant investigational agents prior to definitive surgical resection (post‐treatment). For patients in Nivo±IDO, we used the 5‐week mark for all patients as the post‐treatment sample to standardize the quantity of investigational agents received among patients in this trial. Samples were fractionated via centrifugation. Plasma was collected and stored at −80°C. MILLIPLEX MAP Human Cytokine/Chemokine Magnetic Bead Panels (Millipore) were used to identify cytokines present in plasma at both timepoints. All samples were run in triplicate and median values are presented. Standardized curves were generated for each cytokine, and median fluorescent intensities were transformed into concentrations by 5‐point, non‐linear regression. These concentrations were compared between two groups: patients with and without irAEs. Patients exposed to PD‐1 inhibitors versus PD‐L1 inhibitors were reported separately for this part of the analysis.

### 2.5 Statistical analysis

Descriptive statistics were used for demographic analysis. Categorical data was analyzed using a Chi‐squared or Fisher's exact test. Means were compared using either one‐way ANOVA or Mann–Whitney test. Predictors of irAEs and pathologic treatment response were analyzed by fitting multivariable logistic models using [R]. Backward stepwise variable selection for the multivariable models by Akaike information criterion (AIC) was performed using R package MASS.^23^, ^24^ Kaplan–Meier curves with log‐rank (Mantle‐Cox) tests were used to predict PFS and OS in patients with and without irAEs as well as by p16 status. PFS was defined as the time from study enrollment to recurrence or death from any cause. OS was defined as the time from study enrollment to death from any cause. Patients with missing data for each variable of interest were excluded from the respective analysis. Cytokine levels were compared among groups using a student's *t*‐test. Canonical linear discriminant analysis (LDA) was used to generate predictive cytokine profiles by irAE status.^25^ All analytics were subject to statistical significance level of *p* < 0.05.

## 3 RESULTS

### 3.1 Patient and clinical trial characteristics

Out of 138 patients enrolled across the three clinical trials, 25 were excluded due to trial withdrawal or missing data (Figure S1). A total of 113 patients were included in this study: 38.9% (*n* = 44) Nivo±Tad, 31.9% (*n* = 36) Nivo±IDO, and 29.2% (*n* = 33) Durva±Met. Average follow‐up was 3.0 (±1.4) years. Patient demographics, surgical outcomes, and adjuvant treatment stratified by clinical trial are demonstrated in Table 1.

**TABLE 1:** Patient and clinical trial characteristics.

Mean (SD) or No. of patients (%)	All patients (*n* = 113)	Nivo±Tad (*n* = 44)	Nivo±IDO (*n* = 36)	Durva±Met (*n* = 33)	*p*‐value
Age (years)	61.7 (10.5)	62.5 (10.2)	62.7 (10.3)	59.7 (11.1)	0.414
Sex					
Male	96 (85.0)	41 (93.2)	30 (83.3)	25 (75.8)	0.098
Female	17 (15.0)	3 (6.8)	6 (16.7)	8 (24.2)	
Race					
White	104 (92.0)	40 (90.9)	35 (97.2)	29 (87.9)	0.343
Non‐White	9 (8.0)	4 (9.1)	1 (2.8)	4 (12.1)	
Disease site					
Non‐oropharyngeal	49 (43.4)	20 (45.5)	16 (44.4)	13 (39.4)	0.888
Oropharyngeal	64 (56.6)	24 (54.5)	20 (55.6)	20 (60.6)	
p16‐positive	60 (53.1)	22 (50.0)	17 (47.2)	21 (63.6)	0.339
Recurrent disease	7 (6.2)	2 (4.5)	3 (8.3)	2 (6.1)	0.886
Smoking status					
Never	45 (39.8)	15 (34.1)	15 (41.7)	15 (45.5)	0.205
Former	48 (42.5)	22 (50.0)	11 (30.6)	15 (45.5)	
Current	20 (17.7)	7 (15.9)	10 (27.8)	3 (9.1)	
AJCC 8th edition clinical staging					
I	60 (53.1)	24 (54.5)	15 (41.7)	21 (63.6)	0.348
II	18 (15.9)	4 (9.1)	8 (22.2)	6 (18.2)	
III	7 (6.2)	3 (6.8)	3 (8.3)	1 (3.0)	
IV A/B	28 (24.8)	13 (29.5)	10 (27.8)	5 (15.2)	
Clinical T‐stage					
T0	2 (1.8)	0 (0)	0 (0)	2 (6.1)	0.460
T1	39 (34.5)	14 (31.8)	12 (33.3)	13 (39.4)	
T2	44 (38.9)	18 (40.9)	14 (38.9)	12 (36.4)	
T3	12 (10.6)	3 (6.8)	5 (13.9)	4 (12.1)	
T4a	16 (14.2)	9 (20.5)	5 (13.9)	2 (6.1)	
Clinical N‐stage					
N0	32 (28.3)	10 (22.7)	11 (30.6)	11 (33.3)	0.808
N1	57 (50.4)	25 (56.8)	17 (47.2)	15 (45.5)	
N2	8 (7.1)	3 (6.8)	3 (8.3)	2 (6.1)	
N2a	4 (3.5)	3 (6.8)	1 (2.8)	0 (0)	
N2b	4 (3.5)	1 (2.3)	1 (2.8)	2 (6.1)	
N2c	7 (6.2)	1 (2.3)	3 (8.3)	3 (9.1)	
N3	1 (0.9)	1 (2.3)	0 (0)	0 (0)	
Pathologic T‐stage					
Tx	1 (0.9)	1 (2.3)	0 (0)	0 (0)	0.557
Tis	1 (0.9)	0 (0)	1 (2.8)	0 (0)	
T0	10 (8.8)	3 (6.8)	6 (16.7)	1 (3.0)	
T1	35 (31.0)	14 (31.8)	10 (27.8)	11 (33.3)	
T2	33 (29.2)	13 (29.5)	8 (22.2)	12 (36.4)	
T3	10 (8.8)	3 (6.8)	3 (8.3)	4 (12.1)	
T4a	22 (19.5)	10 (22.7)	8 (22.2)	4 (12.1)	
T4b	1 (0.9)	0 (0)	0 (0)	1 (3.0)	
Pathologic N‐stage					
Nx	3 (2.7)	0 (0)	1 (2.8)	2 (6.1)	0.292
N0	34 (30.1)	10 (22.7)	14 (38.9)	10 (30.3)	
N1	50 (44.2)	22 (50.0)	14 (38.9)	14 (42.4)	
N2	7 (6.2)	4 (9.1)	1 (2.8)	2 (6.1)	
N2a	4 (3.5)	4 (9.1)	0 (0)	0 (0)	
N2b	9 (8.0)	3 (6.8)	3 (8.3)	3 (9.1)	
N3	3 (2.7)	1 (2.3)	2 (5.6)	0 (0)	
N3b	3 (2.7)	0 (0)	1 (2.8)	2 (6.1)	
Lymphovascular invasion (*n* = 103^a^)	22 (21.4)	8 (19.5)	5 (16.7)	9 (28.1)	0.541
Perineural invasion (*n* = 103)^a^	30 (29.1)	11 (26.8)	8 (26.7)	11 (34.4)	0.763
Extracapsular extension (*n* = 75)^b^					0.132
Microscopic	7 (9.3)	1 (3.0)	1 (4.8)	5 (2.4)	
Gross	8 (10.7)	5 (15.2)	2 (9.5)	1 (4.8)	
Positive margins (*n* = 103^a^)	2 (1.9)	1 (2.4)	1 (3.3)	0 (0)	0.756
Adjuvant treatment					
None^c^	43 (38.1)	19 (43.2)	15 (41.2)	9 (27.3)	0.545
Radiation alone	45 (39.8)	15 (34.1)	13 (36.1)	17 (51.5)	
Radiation and chemotherapy	25 (22.1)	10 (22.7)	8 (22.2)	7 (21.2)	
Follow‐up duration (years)	3.0 (1.4)	3.7 (1.6)	2.1 (0.8)	2.9 (1.0)	<0.001

Abbreviations: AJCC, American Joint Committee on Cancer Criteria; IDO, indoleamine 2,3‐dioxygenase; SD, standard deviation.

^a^ Patients with pathologic T0 or Tx disease were excluded from counts as there was no primary tumor identified.

^b^ Patients with greater than pathologic N0 disease.

^c^ Of this group, 18 (37.5%) were recommended but refused adjuvant treatment.

### 3.2 Immune‐related adverse events

Thirty‐two (28.3%) patients developed irAEs of any grade during their treatment or follow‐up (Table 2). Two patients (1.8%) developed Grade 3 irAEs, both in Nivo‐IDO (colitis and hepatitis). The remainder of irAEs were Grade 1–2. Average time from initiation of treatment to irAE onset was 48.3 (±42.8) days. On multivariable analysis, positive p16 status was significantly associated with irAEs (odds ratio [OR] 2.489; 95% CI 1.069–6.119; *p* = 0.043). There was no association between age, sex, race, smoking status, disease site, randomization group, presence of recurrent disease on enrollment, and the development of irAEs by multivariable logistic regression. Clinical stage as a predictor was included in the multivariable model for endpoint irAE. This predictor was retained in the backward variable selection algorithms as determined by AIC but needed to be manually removed due to the mal‐estimates related to the limited sample size. When comparing patients without clinical nodal involvement to those with clinical nodal involvement, there was no significant difference in rate of irAE (OR 0.488; 95% CI 0.1811–1.342; *p* = 0.174).

**TABLE 2:** Frequency of immune‐related adverse events by clinical trial.

No. of patients (%)	All patients (*n* = 113)	Nivo±Tad (*n* = 44)	Nivo±IDO (*n* = 36)	Durva±Met (*n* = 33)	*p*‐value
irAE	32 (28.3)	11 (25.0)	12 (33.3)	9 (27.3)	0.700
Dermatologic	17 (15.0)	6 (13.6)	4 (11.1)	7 (21.2)	0.538
Dermatitis	16 (14.1)	6 (13.6)	4 (11.1)	6 (18.2)	
Psoriasis flare	1 (0.9)	–	–	1 (3.0)	
Mucosal	1 (0.9)	–	1 (2.8)	–	0.611
Mucositis	1 (0.9)	0	1 (2.8)	–	
Endocrine	8 (7.1)	2 (4.5)	3 (8.3)	3 (9.1)	0.725
Hypothyroidism	5 (4.4)	2 (4.5)	–	3 (9.1)	
Hyperthyroidism	2 (1.8)	–	2 (5.6)	–	
Adrenal insufficiency	1 (0.9)	–	1 (2.8)	–	
Gastrointestinal	5 (4.4)	2 (4.5)	3 (8.3)	–	0.271
Colitis	1 (0.9)	–	1 (2.8)	–	
Diarrhea	3 (2.7)	2 (4.5)	1 (2.8)	–	
Hepatitis	1 (0.9)	–	1 (2.8)	–	
Musculoskeletal	8 (7.1)	4 (6.8)	3 (8.3)	1 (3.0)	0.653
Myalgia/arthralgia	7 (6.2)	4 (6.8)	2 (5.6)	1 (3.0)	
Arthritis	1 (0.9)	–	1 (2.8)	–	
Multiple irAEs	5 (4.4)	1 (2.3)	2 (5.6)	2 (6.1)	0.620

Abbreviations: IDO, indoleamine 2,3‐dioxygenase; irAE, immune‐related adverse event.

### 3.3 Immune‐related adverse events were associated with greater pathologic response to treatment

Fifty (44.2%) patients demonstrated pathologic treatment response. Within a multivariable model controlling for age, sex, smoking status, disease site, p16 status, randomization group, presence of recurrent disease on enrollment and clinical staging, there was a significant association between presence of irAEs and pathologic treatment response (OR 3.730; 95% CI 1.344–10.350; *p* = 0.011). Within the multivariable model, patients in Durva±Met were less likely to be pathologic responders than those in Nivo±Tad (OR 0.132; 95% CI 0.037–0.469; *p* = 0.002) or in Nivo±IDO (OR 0.076; 95% CI 0.019–0.299; *p* < 0.001). Interestingly, white patients were less likely to be pathologic responders than non‐white patients within the multivariable model (OR 0.138; 95% CI 0.025–0.753; *p* = 0.022). Among patients with quantifiable pTE% (Nivo±Tad and Nivo±IDO), mean pTE% in patients with irAEs was 45.6% versus 26.7% in patients without irAEs (*p* = 0.018).

### 3.4 Survival benefit in patients with immune‐related adverse events

**FIGURE 1:**
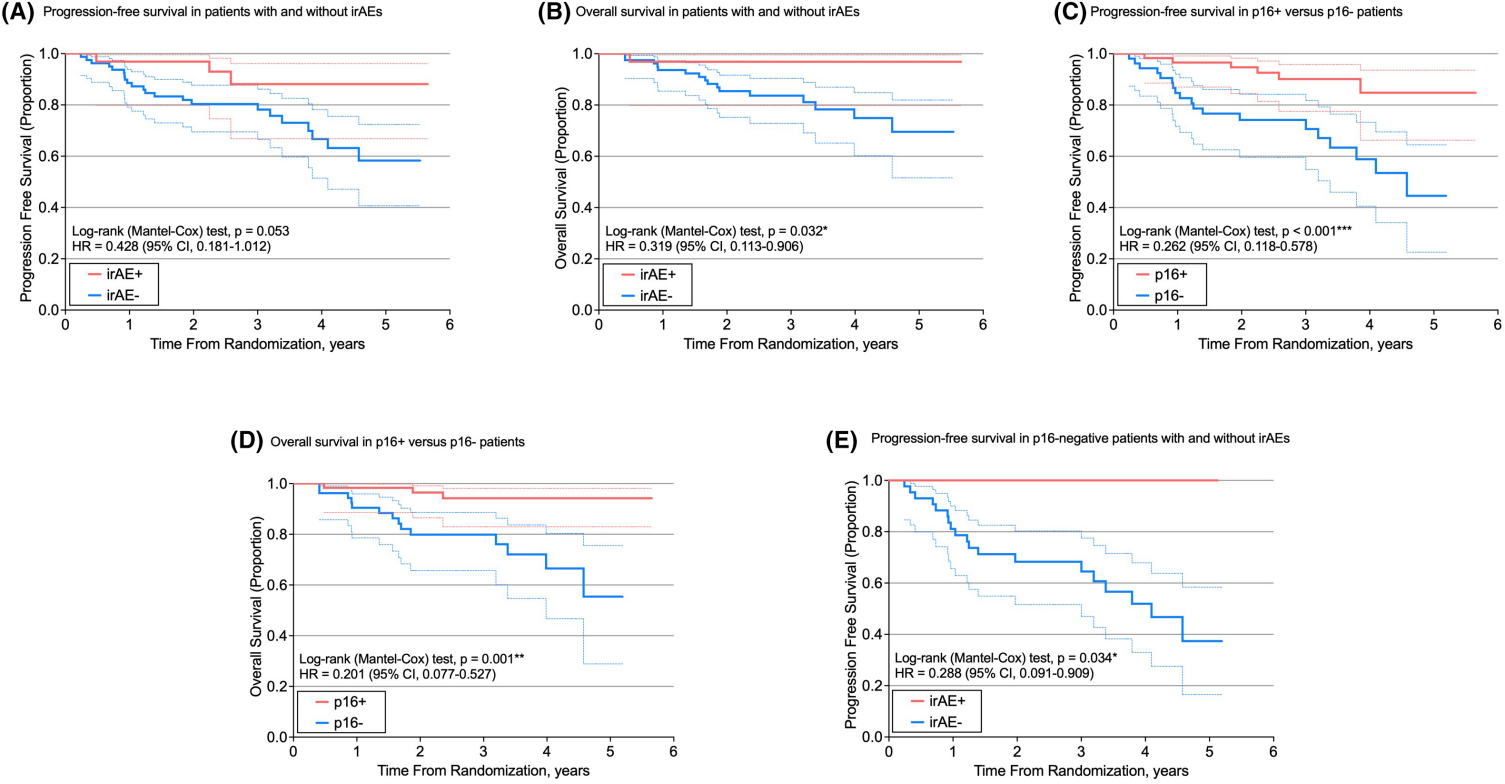
Kaplan–Meier survival plots. Compared to patients without irAEs, patients with irAEs demonstrated (A) a higher progression‐free survival (PFS) (approaching significance with a *p*‐value of 0.053) and (B) a significantly higher overall survival (OS) (*p* = 0.032). Patients with p16‐positive tumors had a significantly higher (C) PFS (*p* < 0.001) and (D) OS (*p* = 0.001) compared to patients with p16‐negative tumors. (E) In the subset of p16‐negative patients, patients with irAEs had a significantly higher PFS (*p* = 0.034). irAE, immune‐related adverse event; OS, overall survival; PFS, progression‐free survival.

Kaplan–Meier survival curves were generated for PFS and OS (Figure 1). IrAEs were associated with a significantly higher OS (hazard ratio [HR] 0.319; 95% CI 0.113–0.906; *p* = 0.032) as well as a higher PFS, approaching significance (HR 0.428; 95% CI 0.181–1.012; *p* = 0.053). When stratified by p16 status, p16‐negative patients with irAEs demonstrated a significantly higher PFS than those without (HR 0.288; 95% CI 0.091–0.909; *p* = 0.034). Compared to patients with p16‐negative tumors, patients with p16‐positive tumors demonstrated a significantly higher PFS (HR 0.262; 95% CI 0.118–0.578; *p* < 0.001) and OS (HR 0.201; 95% CI 0.077–0.527; *p* = 0.001). Within trials, there was no statistically significant difference in PFS or OS between randomization groups. PFS and OS did not vary between trials.

### 3.5 Elevated concentration of circulating cytokines in patients with immune‐related adverse events

**FIGURE 2:**
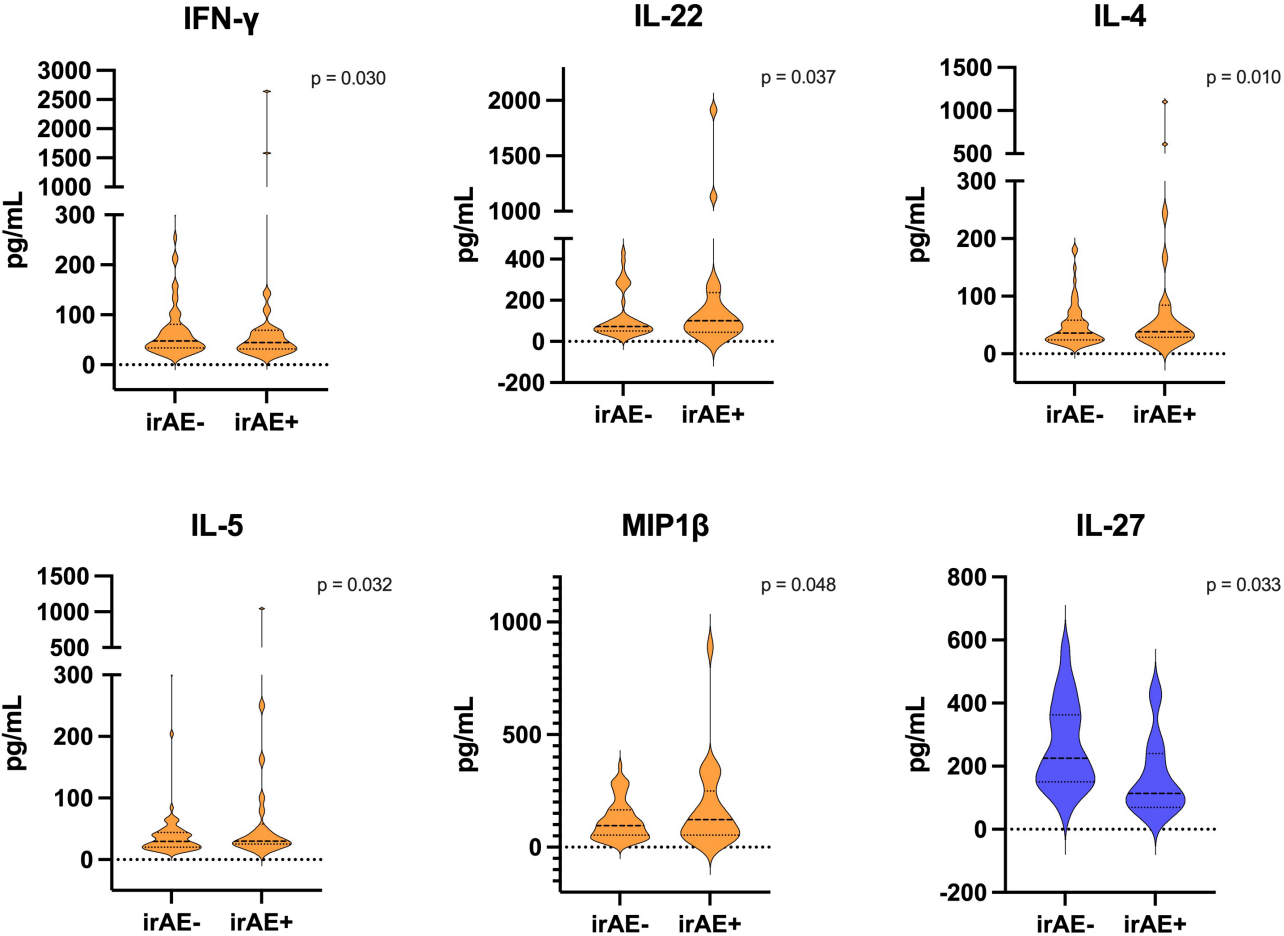
Significant pre‐treatment cytokine differences in patients with and without irAEs. Pre‐treatment cytokines with significant concentration (pg/mL) differences among irAE‐ and irAE+ are demonstrated (orange: Nivo±Tad and Nivo±IDO, blue: Durva±Met). IFN‐γ, interferon‐gamma; IL, interleukin; irAE, immune‐related adverse event; MIP1β, Macrophage inflammatory protein‐1 beta.

The concentrations (pg/mL) of circulating cytokines were compared between patients with and without irAEs in Nivo±Tad and Nivo±IDO (Table 3). Within Nivo±Tad and Nivo±IDO, patients with irAEs had significantly higher pre‐treatment concentrations of MIP1β, interferon‐gamma (IFN‐γ), interleukin (IL)4, IL5, and IL22 (Figure 2). Patients with dermatologic irAEs within Nivo±Tad and Nivo±IDO had significantly higher pre‐treatment concentrations of fractalkine, GMCSF, IFN‐γ, IL22, IL25, IL27, IL4, IL5, IL6, MIG, MIP1β, tumor necrosis factor‐alpha (TNF‐α), and TNF‐β. Within Durva±Met, patients with irAEs had a significantly lower pre‐treatment concentration of IL27 (*p* = 0.033). There were no cytokine differences in Durva±Met when looking only at patients with dermatologic irAEs compared to the remainder of the Durva±Met study population. In addition, compared to non‐responders without irAEs, responders with irAEs had significantly higher pre‐treatment concentrations of IL4 (*p* = 0.04) and MIP1β (*p* = 0.036). The full list of cytokines analyzed as well as additional comparisons are provided in Table S1.

**TABLE 3:** Comparing mean concentration of circulating cytokines (pg/mL) between irAE+ and irAE‐ patients treated with nivolumab.

	Pre‐treatment irAE+/irAE‐		Pre‐treatment irAE+/irAE‐ (dermatologic)	
	Δ	*p*‐value	Δ	*p*‐value
Fractalkine	–	0.225	↑	0.022
GMCSF	–	0.149	↑	0.013
IFN‐γ	↑	0.030	↑	<0.001
IL22	↑	0.037	↑	0.001
IL25	–	0.177	↑	0.015
IL27	–	0.076	↑	0.001
IL4	↑	0.010	↑	<0.001
IL5	↑	0.032	↑	<0.001
IL6	–	0.296	↑	0.05
MIG	–	0.059	↑	0.011
MIP1β	↑	0.048	↑	0.014
TNF‐α	–	0.194	↑	0.008
TNF‐β	–	0.231	↑	0.027

Abbreviations: GMCSF, Granulocyte‐macrophage colony‐stimulating factor; IFN‐γ, interferon‐gamma; IL, interleukin; irAE, immune‐related adverse event; MIG, Monokine induced by gamma; MIP1β, Macrophage inflammatory protein‐1 beta; TNF, tumor necrosis factor.

### 3.6 Pre‐treatment cytokine profiles predict immune‐related adverse events

**FIGURE 3:**
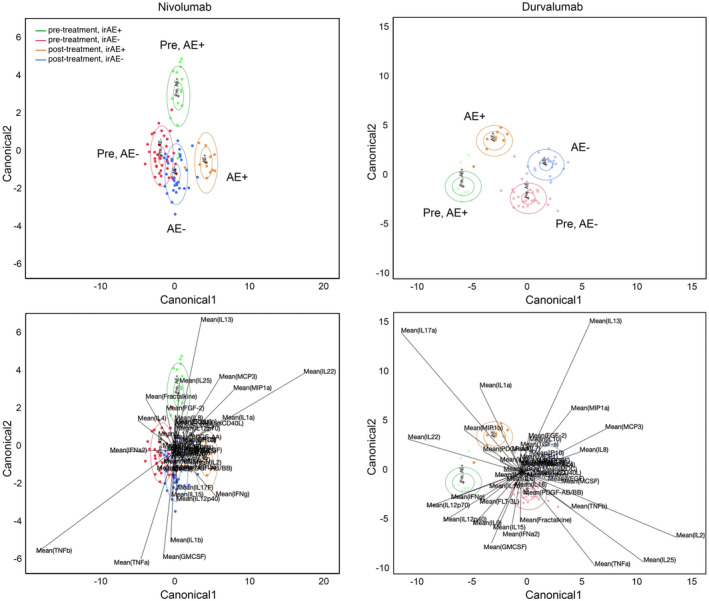
Canonical linear discriminate analysis (LDA) of cytokine profiles. 95% confidence level ellipse (smaller, inside) and 50% confidence level ellipse (larger, outside) represent samples in associated categories. Biplot rays indicate the directions of the predictors in the canonical space. Cytokine profiling was predictive of future development of irAEs within Durva±Met and nivolumab samples. IrAE+ patients were accurately predicted 100% (9/9) of the time in Durva±Met and 82% (13/16) in nivolumab samples (green ellipse). IrAE‐ patients were accurately predicted 91% (21/23) of the time in Durva±Met and 89% (33/37) in nivolumab samples (red ellipse). irAE, immune‐related adverse event.

On canonical LDA, there were evident differences in pre‐treatment cytokine profiles between patients who went on to develop irAEs and those who did not (Figure 3). Within Nivo±Tad and Nivo±IDO, pre‐treatment canonical LDA accurately identified 82% (13/16) of future irAE+ patients (green ellipse) and 89% (33/37) of future irAE‐ patients (red ellipse). Key drivers of irAEs within the nivolumab model included IL1α, IL13, IL22, IL25, CCL7 (MCP3), CCL3 (MIP1α), and IFN‐γ. Similarly in Durva±Met, pre‐treatment canonical LDA accurately identified future irAE+ patients 100% (9/9) of the time (green ellipse) and future irAE‐ patients 91% (21/23) of the time (red ellipse). Key drivers of irAEs within the Durva±Met model included IL1α, IL9, IL22, IL17, IL12p40, IL12p70, and IFN‐γ. Within both models, patients without irAEs had less change in cytokine activity from pre‐ to post‐treatment, evidenced by proximity of red (pre‐treatment, irAE‐) and blue (post‐treatment, irAE‐) ellipses in Figure 3.

## 4 DISCUSSION

With the growing use of ICIs comes the importance of balancing treatment efficacy with associated side effects that may indicate therapeutic advantage. The immune system is tightly regulated to protect against autoimmunity. As ICIs lift this protection through disinhibition of immune checkpoints, it is important to anticipate both antitumor and autoimmune effects, including the development of irAEs. Studies have suggested that development of irAEs represents an enhanced T‐cell‐mediated immunoreaction and could, therefore, indicate enhanced response to ICIs.^5^, ^6^, ^7^, ^11^ A recent review by Kessler et al. underscored the scarcity of literature evaluating irAEs in the setting of HNSCC. Within their review, they identified only three studies that directly related irAEs to favorable outcomes in patients with HNSCC undergoing treatment with ICIs.^26^, ^27^, ^28^, ^29^ Outside of this study, only a few others have identified similar associations.^30^, ^31^

In this study, we found that irAEs were associated with improved oncologic outcomes in HNSCC, consistent with previously reported literature. While treated in different settings and with different modalities, HNSCC patients with irAEs were more likely to be pathologic responders and have a higher OS (*p* = 0.037) compared to those without irAEs. In a recent systematic review and meta‐analysis, Zhou et al. evaluated 30 studies and found that patients who developed irAEs secondary to ICIs experienced both an OS benefit and a PFS benefit. This was particularly true in patients with endocrine and dermatologic irAEs as well as low‐grade irAEs, which is consistent with the findings of our current study.^7^

No patients in this cohort had greater than Grade 3 irAEs, with a majority being Grade 1–2. Two patients in Nivo±IDO developed Grade 3 irAEs (hepatitis and colitis), both of which had already completed the study intervention. Following the development of hepatitis, the patient was treated with a long taper of high‐dose prednisone. Liver function tests normalized after 6 months of steroids and have remained within normal limits as of most recent follow‐up. The second patient developed Grade 3 colitis, ultimately diagnosed with Crohn's disease that was potentially unmasked by his immunotherapy treatment, as well as Grade 2 synovitis involving multiple joints that was negative for RF, ANA, CCP, and ANCA. This patient was started on prednisone and infliximab and has had good disease control as of last follow‐up. Both patients were pathologic responders (pTE 50% and 63%, respectively) and have been without evidence of disease as of most recent follow‐up. Our data suggests that Grade 1–3 irAEs could represent greater immune competence and response to therapy and should not deter providers or patients from continuing ICI therapy. In addition, Paderi et al. demonstrated that early treatment (before 30 days) with systemic corticosteroids in patients with irAEs secondary to ICIs did not significantly affect PFS in NSCLC, melanoma, and renal cell carcinoma. Further, patients with irAEs who were treated with systemic corticosteroids after 30 days demonstrated a significantly longer PFS.^32^ Further evidence is necessary before recommending steroid treatment in patients with irAEs, but this data suggests a potential use of steroids in patients with higher‐grade irAEs to balance treatment efficacy with systemic side effects.

We found a significant association between positive p16 status and the presence of irAEs (OR 2.489; 95% CI 1.069–6.119; *p* = 0.043). To the authors' knowledge, this association has not been described in prior literature. HPV‐mediated tumors arise in an immune‐rich environment; therefore, one would expect ICIs to exacerbate immune reactions in these patients.^33^ One hypothesis for the pathogenesis of irAEs may relate to the tumor‐host interactions. Tumor‐induced inflammation could also explain elevated proinflammatory pre‐treatment cytokines predisposing patients to irAEs.^2^ As positive p16 status is a favorable prognostic factor, this may also represent bias for the clinical implications of irAEs. To control for this bias, we conducted a multivariable logistic regression for pathologic treatment response, demonstrating a significant association between the presence of irAEs and pathologic treatment response (OR 3.730; 95% CI 1.344–10.350; *p* = 0.011) while controlling for possible confounding variables, including p16 status. In contrast to our finding, the Checkmate 358 trial led by Ferris et al. noted a similar incidence of select treatment‐related adverse events (which they defined as adverse events with potential immunologic cause) between HPV‐positive and HPV‐negative cohorts following neoadjuvant treatment with nivolumab for HNSCC.^34^ Further studies are needed to investigate the possible relationship between p16/HPV status and development of irAEs following ICI therapy.

While the link between irAEs and treatment efficacy has been well‐documented in current literature, there is a significant gap in research identifying predictive biomarkers for the development of irAEs. Several molecular biomarkers have been suggested, such as high baseline C‐reactive protein or soluble cytotoxic T‐lymphocyte‐associated antigen‐4 (CTLA‐4).^35^, ^36^, ^37^ In melanoma patients treated with anti‐PD‐1 and/or anti‐CTLA‐4 immunotherapy, Lim et al. reported that elevation of an 11‐cytokine assay, which they termed the CYTOX score, can predict severe toxicity from ICIs.^2^, ^20^ Their finding of elevated cytokines in pre‐treatment samples is consistent with a previous hypothesis that irAEs represent subclinical inflammation that is then triggered by ICI therapy.^2^ Further, T‐cell exhaustion has been studied extensively and noted to be a major obstacle in formulating effective immunotherapeutics as blunted T‐cell effector function impacts the body's ability to mount an appropriate immune response.^38^, ^39^, ^40^, ^41^ We speculated that patients who go on to develop irAEs are likely to have higher baseline immune fitness as determined by higher baseline levels of proinflammatory cytokines. Using Luminex multiplex assays performed on pre‐treatment peripheral blood samples, we found significant differences in cytokine profiles between patients who experienced irAEs and those who did not within the two nivolumab trials. Higher baseline concentrations of MIP1β, IFN‐γ, IL4, IL5, and IL22 were associated with the development of irAEs from any category. When stratified by patients with dermatologic irAEs, higher concentrations of fractalkine, GMCSF, IFN‐γ, IL22, IL25, IL27, IL4, IL5, IL6, MIG, MIP1β, TNF‐α, and TNF‐β were associated with development of dermatologic irAEs. Many of these cytokines have been described as key players in inflammatory and autoimmune processes, as hypothesized.^42^, ^43^, ^44^, ^45^, ^46^, ^47^

While several studies have evaluated the association between baseline peripheral cytokine levels as well as changes in cytokine levels over time as predictors of response to immunotherapy agents and development of irAEs in various cancers, a clear correlation has not yet been determined.^48^, ^49^, ^50^, ^51^, ^52^ In this study, we used canonical LDA to identify combinations of cytokines that can be used to predict the development of irAEs. Canonical LDA, first described by Rao, is a method of dimensionality reduction and classification derived from the Fisher LDA. This method of statistical analysis involves utilizing pattern recognition and machine learning to discriminate between groups by identifying patterns based on the linear combinations of multiple variables.^25^, ^53^ LDA has multiple applications including pattern recognition, facial recognition, and bioinformatics. It has been studied in various cohorts, including immunotherapy interventions, to identify predictive cytokine profiles.^54^, ^55^, ^56^, ^57^, ^58^ Using canonical LDA, we identified cytokine profiles that were highly predictive for future irAE+ and irAE‐ subjects. Our model identified key drivers of irAEs within Durva±Met (IL1α, IL9, IL22, IL17, IL12p40, IL12p70, and IFN‐γ) and nivolumab trials (IL1α, IL13, IL22, IL25, CCL7 (MCP3), CCL3 (MIP1α), and IFN‐γ). This powerful tool demonstrates the utility of pre‐treatment cytokine testing to reliably predict future irAE status of patients receiving immunotherapy. Still, further validation is necessary to confirm these key drivers of irAEs.

In our cytokine analysis, we presented Durva±Met separately due to its different mechanism of action from nivolumab. On analysis of Durva±Met, we did not see the same pattern of cytokine elevation or as significant of an effect on circulating cytokine concentrations over time as we saw with nivolumab. As durvalumab's mechanism of inhibition is different from nivolumab's, we suspect that the development of irAEs in these patients may proceed through a different pathway with different cytokines at play.

Although documentation was collected prospectively for each trial, a major limitation of this retrospective study is that the comparison of these cohorts was not powered for these endpoints. A prospective study design would better control for possible bias and confounding. A larger sample size in a multi‐institutional prospective study would allow for greater generalizability, more extensive validation, and a better understanding of the relationship between circulating cytokines, irAEs, and response to neoadjuvant ICIs in patients with HNSCC.

## 5 CONCLUSIONS

irAEs, even at a moderate level, are associated with improved oncologic outcomes in HNSCC patients treated with neoadjuvant ICIs administered in different combinations and therapeutic schedules. irAEs may represent greater immune competence and, as a result, greater antitumor efficacy of ICIs. In this cohort, irAEs secondary to nivolumab were characterized by elevation of select circulating cytokines. Canonical LDA identified sets of circulating cytokines that could be used to predict the development of irAEs. The ability to predict and manage irAEs early may help maximize the therapeutic benefit of neoadjuvant ICIs in patients with HNSCC and manage patient expectations.

## AUTHOR CONTRIBUTIONS

**Angela E. Alnemri:** Conceptualization (lead); data curation (lead); formal analysis (lead); investigation (lead); methodology (lead); project administration (lead); resources (lead); software (lead); supervision (equal); validation (lead); visualization (lead); writing – original draft (lead); writing – review and editing (lead). **Sruti Tekumalla:** Data curation (equal); investigation (equal); resources (equal); writing – original draft (equal); writing – review and editing (equal). **Annie E. Moroco:** Investigation (equal); methodology (equal); supervision (equal); validation (equal); visualization (equal); writing – review and editing (equal). **Ioannis Vathiotis:** Conceptualization (equal); formal analysis (equal); investigation (equal); methodology (equal); supervision (equal); writing – review and editing (equal). **Madalina Tuluc:** Data curation (equal); methodology (supporting); validation (equal); writing – original draft (supporting); writing – review and editing (supporting). **Stacey Gargano:** Data curation (equal); methodology (supporting); validation (equal); writing – original draft (supporting); writing – review and editing (supporting). **Tingting Zhan:** Formal analysis (lead); software (lead); validation (lead); writing – review and editing (supporting). **David M. Cognetti:** Conceptualization (equal); methodology (equal); supervision (equal); validation (equal); writing – review and editing (equal). **Joseph M. Curry:** Conceptualization (equal); methodology (equal); supervision (equal); validation (equal); writing – review and editing (equal). **Athanassios Argiris:** Conceptualization (equal); methodology (equal); supervision (equal); validation (equal); writing – review and editing (equal). **Alban Linnenbach:** Conceptualization (equal); investigation (equal); methodology (equal); supervision (equal); validation (equal); writing – review and editing (equal). **Andrew P. South:** Investigation (equal); methodology (equal); supervision (equal); validation (equal); writing – review and editing (equal). **Larry A. Harshyne:** Conceptualization (lead); data curation (lead); formal analysis (equal); investigation (equal); methodology (equal); software (lead); supervision (lead); writing – original draft (equal); writing – review and editing (lead). **Jennifer M. Johnson:** Conceptualization (lead); investigation (equal); methodology (equal); project administration (equal); supervision (lead); validation (equal); writing – review and editing (lead). **Adam J. Luginbuhl:** Conceptualization (lead); data curation (equal); investigation (lead); methodology (lead); project administration (lead); supervision (lead); validation (lead); visualization (lead); writing – original draft (lead); writing – review and editing (lead).

## FUNDING INFORMATION

The data analyzed in this study came from clinical trials funded by Bristol‐Myers Squibb (NCT03238365, NCT03162731) and AstraZeneca (NCT03618654). This research was independent of the trial protocols and was not specifically funded. This study utilized the Biostatistics Shared Resource at the Sidney Kimmel Cancer Center—Jefferson Health, supported by the NCI, grant 5P30CA056036‐18.

## CONFLICT OF INTEREST STATEMENT

D.M. Cognetti has received grants from Bristol‐Myers Squibb during the conduct of the clinical trials reported and personal fees from Rakuten Medical Inc., Intuitive, and Cortexyme outside the submitted work. J.M. Curry has received nonfinancial support from AstraZeneca and personal fees from Rakuten Medical Inc. outside the submitted work. A. Argiris is on the advisory board, Speakers' Bureau, and has received research funding from Bristol‐Myers Squibb. A.P. South has received research funding and consults for Oncononva Therapeutics, and owns stock in Krystal Biotech Inc. J.M. Johnson has received research funding from Bristol‐Myers Squibb and AstraZeneca. A.J. Luginbuhl has received grants from Bristol‐Myers Squibb during the conduct of the clinical trials reported. No disclosures were reported by the other authors.

## ETHICAL APPROVAL STATEMENT

All clinical trials had unique full approval by the Thomas Jefferson University Institutional Review Board. This retrospective study was determined to be exempt from full review (IRB #21E.431).

## PATIENT CONSENT STATEMENT

This retrospective study was determined to be exempt from requiring written informed consent for use of patient materials by the Thomas Jefferson University Institutional Review Board (IRB #21E.431).

## Supporting information


**Data S1:** Supporting Information.

## Data Availability

The data that support the findings of this study are available from the corresponding author upon reasonable request.
