# Supplementary material for: Predictive capacity of immune‐related adverse events and cytokine profiling in neoadjuvant immune checkpoint inhibitor trials for head and neck squamous cell carcinoma
Source: Cancer Med. 2024 Jun 7;13(11):e7370. doi: 10.1002/cam4.7370 (PMC11157197; doi:10.1002/cam4.7370)
Supplement: Supplementary file 1 — Data S1: Supporting Information. [file CAM4-13-e7370-s001.docx]

**Supplementary Figure 1**. Trial inclusion and exclusion.


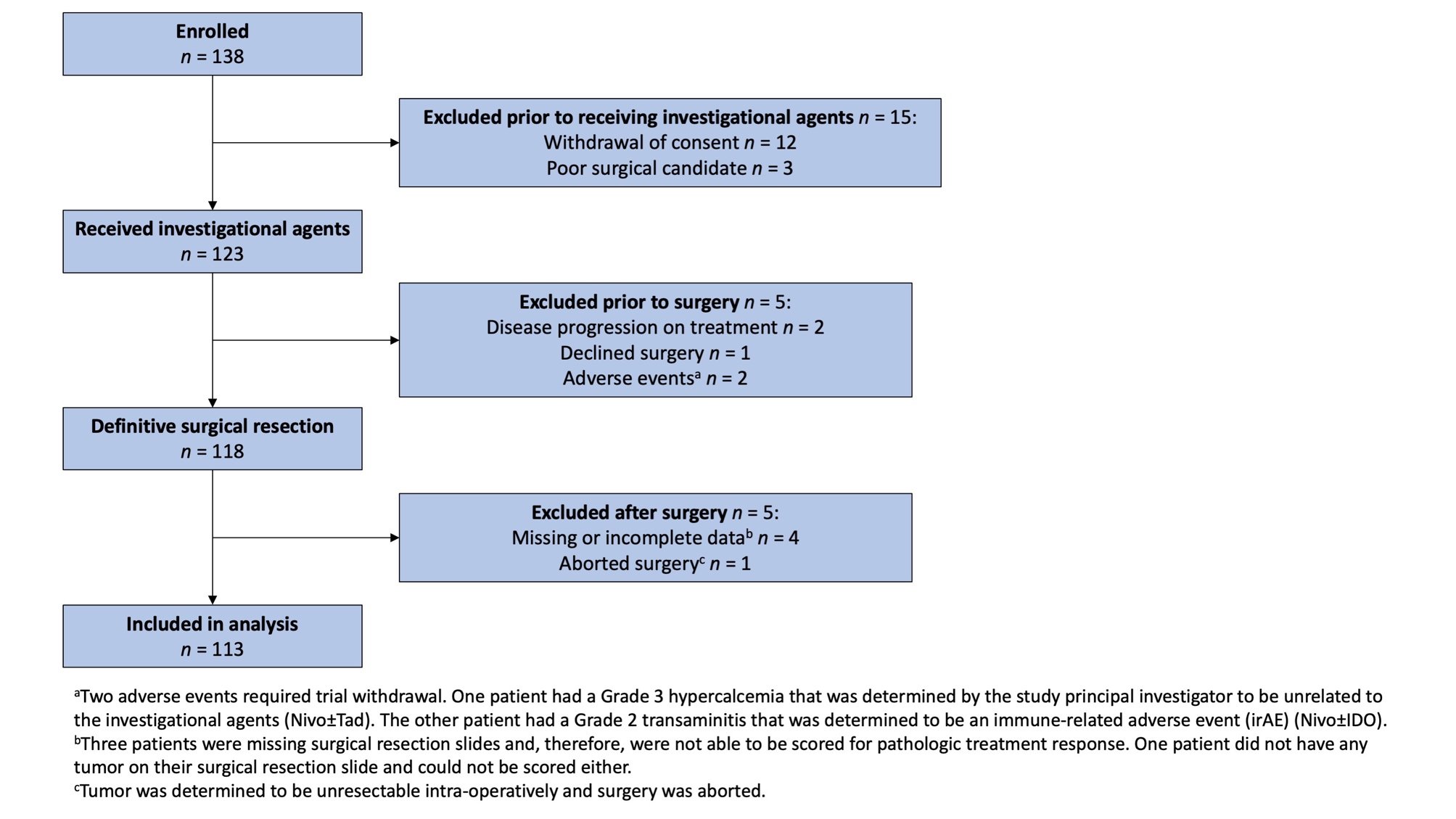


**Supplementary Table 1**. Full set of cytokines analyzed.

|  | Nivo Pre-tx irAE+/irAE- | p-value | Durva Pre-tx irAE+/irAE- | p-value | Nivo Pre-tx Derm irAE+/irAE- | p-value | Durva Pre-tx Derm irAE+/irAE- | p-value | Pre-tx irAE+, R/irAE-, NR | p-value | Nivo post-tx/pre-tx irAE+ | Nivo post-tx/pre-tx irAE- | p-value | Durva post-tx/pre-tx irAE+ | Durva post-tx/pre-tx irAE- | p-value |
| --- | --- | --- | --- | --- | --- | --- | --- | --- | --- | --- | --- | --- | --- | --- | --- | --- |
| EGF |  | 0.350 |  | 0.352 |  | 0.142 |  | 0.123 |  | 0.458 |  |  | 0.601 |  |  | 0.188 |
| Eotaxin |  | 0.128 |  | 0.226 |  | 0.200 |  | 0.135 |  | 0.441 |  |  | 0.125 |  |  | 0.320 |
| FGF-2 |  | 0.251 |  | 0.365 |  | 0.133 |  | 0.169 |  | 0.333 |  |  | 0.278 |  |  | 0.807 |
| FLT-3L |  | 0.199 |  | 0.475 |  | 0.393 |  | 0.170 |  | 0.315 |  |  | 0.568 |  |  | 0.807 |
| Fractalkine |  | 0.225 |  | 0.284 | ↑ | 0.022 |  | 0.180 |  | 0.296 |  |  | 0.770 |  |  | 0.622 |
| GCSF |  | 0.491 |  | 0.410 |  | 0.394 |  | 0.213 |  | 0.127 |  |  | 0.317 |  |  | 0.979 |
| GMCSF |  | 0.149 |  | 0.182 | ↑ | 0.013 |  | 0.220 |  | 0.293 |  |  | 0.752 |  |  | 0.468 |
| GROa |  | 0.280 |  | 0.453 |  | 0.324 |  | 0.223 |  | 0.211 |  |  | 0.317 |  |  | 0.938 |
| IFNa2 |  | 0.367 |  | 0.470 |  | 0.427 |  | 0.236 |  | 0.291 |  |  | 0.562 |  |  | 0.596 |
| IFNg | ↑ | 0.030 |  | 0.345 | ↑ | 0.000 |  | 0.245 |  | 0.066 |  |  | 0.646 |  |  | 0.577 |
| IL10 |  | 0.340 |  | 0.353 |  | 0.295 |  | 0.253 |  | 0.442 | ↑ | ↑ | 0.016 |  |  | 0.929 |
| IL12p40 |  | 0.478 |  | 0.439 |  | 0.173 |  | 0.259 |  | 0.184 |  |  | 0.745 |  |  | 0.897 |
| IL12p70 |  | 0.479 |  | 0.316 |  | 0.381 |  | 0.262 |  | 0.477 |  |  | 0.364 |  |  | 0.669 |
| IL13 |  | 0.462 |  | 0.474 |  | 0.176 |  | 0.279 |  | 0.307 |  |  | 0.985 |  |  | 0.835 |
| IL15 |  | 0.306 |  | 0.295 |  | 0.161 |  | 0.284 |  | 0.254 |  |  | 0.159 |  |  | 0.713 |
| IL17a |  | 0.401 |  | 0.424 |  | 0.499 |  | 0.300 |  | 0.240 |  |  | 0.385 |  |  | 0.853 |
| IL17F |  | 0.188 |  | 0.331 |  | 0.368 |  | 0.307 |  | 0.182 |  |  | 0.361 |  |  | 0.927 |
| IL18 |  | 0.159 |  | 0.253 |  | 0.259 |  | 0.316 |  | 0.310 | ↓ | ↓ | 0.022 | ↓ | ↓ | 0.028 |
| IL1a |  | 0.259 |  | 0.452 |  | 0.398 |  | 0.324 |  | 0.196 |  |  | 0.293 |  |  | 0.722 |
| IL1b |  | 0.423 |  | 0.479 |  | 0.344 |  | 0.338 |  | 0.314 |  |  | 0.582 |  |  | 0.766 |
| IL1RA |  | 0.182 |  | 0.209 |  | 0.498 |  | 0.341 |  | 0.174 |  |  | 0.326 | ↓ | ↓ | 0.045 |
| IL2 |  | 0.291 |  | 0.335 |  | 0.494 |  | 0.347 |  | 0.353 |  |  | 0.293 |  |  | 0.728 |
| IL22 | ↑ | 0.037 |  | 0.408 | ↑ | 0.001 |  | 0.349 |  | 0.095 |  |  | 0.535 |  |  | 0.834 |
| IL25 |  | 0.177 |  | 0.410 | ↑ | 0.015 |  | 0.351 |  | 0.227 |  |  | 0.889 |  |  | 0.841 |
| IL27 |  | 0.076 | ↓ | 0.033 | ↑ | 0.001 |  | 0.352 |  | 0.078 | ↑ |  | 0.003 |  |  | 0.093 |
| IL3 |  | 0.300 |  | 0.316 |  | 0.051 |  | 0.354 |  | 0.285 |  |  | 0.651 |  |  | 0.368 |
| IL4 | ↑ | 0.010 |  | 0.489 | ↑ | 0.000 |  | 0.356 | ↑ | 0.040 |  |  | 0.794 |  |  | 0.969 |
| IL5 | ↑ | 0.032 |  | 0.361 | ↑ | 0.000 |  | 0.377 |  | 0.352 |  |  | 0.907 |  |  | 0.699 |
| IL6 |  | 0.296 |  | 0.312 | ↑ | 0.050 |  | 0.378 |  | 0.179 |  |  | 0.627 |  |  | 0.637 |
| IL7 |  | 0.078 |  | 0.251 |  | 0.372 |  | 0.385 |  | 0.289 |  |  | 0.926 |  |  | 0.225 |
| IL8 |  | 0.328 |  | 0.380 |  | 0.354 |  | 0.403 |  | 0.135 |  |  | 0.545 |  |  | 0.801 |
| IL9 |  | 0.141 |  | 0.429 |  | 0.137 |  | 0.408 |  | 0.265 |  |  | 0.478 |  |  | 0.971 |
| IP10 |  | 0.492 |  | 0.484 |  | 0.452 |  | 0.416 |  | 0.446 | ↑ | ↑ | 0.005 | ↑ |  | 0.014 |
| MCP1 |  | 0.363 |  | 0.309 |  | 0.488 |  | 0.424 |  | 0.183 | ↑ | ↑ | 0.032 | ↑ | ↑ | 0.023 |
| MCP3 |  | 0.392 |  | 0.341 |  | 0.337 |  | 0.426 |  | 0.237 |  |  | 0.933 |  |  | 0.615 |
| MCSF |  | 0.407 |  | 0.269 |  | 0.143 |  | 0.427 |  | 0.483 |  |  | 0.640 |  |  | 0.622 |
| MDC |  | 0.404 |  | 0.220 |  | 0.398 |  | 0.427 |  | 0.482 |  |  | 0.993 |  |  | 0.456 |
| MIG |  | 0.059 |  | 0.253 | ↑ | 0.011 |  | 0.432 |  | 0.457 | ↑ |  | 0.002 | ↑ | ↑ | 0.039 |
| MIP1a |  | 0.341 |  | 0.269 |  | 0.454 |  | 0.432 |  | 0.168 |  |  | 0.576 |  |  | 0.424 |
| MIP1b | ↑ | 0.048 |  | 0.363 | ↑ | 0.014 |  | 0.433 | ↑ | 0.036 |  |  | 0.060 |  |  | 0.883 |
| PDGF-AA |  | 0.273 |  | 0.275 |  | 0.449 |  | 0.456 |  | 0.272 |  |  | 0.580 |  |  | 0.294 |
| PDGF-AB/BB |  | 0.343 |  | 0.294 |  | 0.455 |  | 0.456 |  | 0.423 |  |  | 0.895 |  |  | 0.288 |
| RANTES |  | 0.199 |  | 0.386 |  | 0.495 |  | 0.459 |  | 0.161 |  |  | 0.900 |  |  | 0.061 |
| sCD40L |  | 0.216 |  | 0.464 |  | 0.166 |  | 0.467 |  | 0.480 |  |  | 0.351 |  |  | 0.957 |
| TGF-a |  | 0.452 |  | 0.387 |  | 0.320 |  | 0.471 |  | 0.106 |  |  | 0.928 |  |  | 0.839 |
| TNFa |  | 0.194 |  | 0.323 | ↑ | 0.008 |  | 0.480 |  | 0.404 |  |  | 0.649 |  |  | 0.914 |
| TNFb |  | 0.231 |  | 0.370 | ↑ | 0.027 |  | 0.485 |  | 0.462 |  |  | 0.897 |  |  | 0.714 |
